# C-reactive protein induced T cell activation is an indirect monocyte-dependent mechanism involving the CD80/CD28 pathway

**DOI:** 10.3389/fimmu.2025.1622865

**Published:** 2025-07-18

**Authors:** Julia Thomé, Julia Limmer, Teresa Z. Brose, Johannes Zeller, Nina Chevalier, Anna-Lena Schäfer, Laura Schneider, Maike Lind, Thierry Christmann, Marie Dreck, Sheena Kreuzaler, David Braig, Karlheinz Peter, Franziska Pankratz, Steffen U. Eisenhardt

**Affiliations:** ^1^ Department of Plastic and Hand Surgery, Medical Center and Faculty of Medicine, University of Freiburg, Freiburg, Germany; ^2^ Department of Dermatology, Medical Center - University of Freiburg, Freiburg, Germany; ^3^ Department of Rheumatology and Clinical Immunology, Medical Center - University of Freiburg, Faculty of Medicine, University of Freiburg, Freiburg, Germany; ^4^ Center for Chronic Immunodeficiency (CCI), Medical Center - University of Freiburg, Faculty of Medicine, University of Freiburg, Freiburg, Germany; ^5^ Atherothrombosis and Vascular Biology Laboratory, Baker Heart and Diabetes Institute, Melbourne, VIC, Australia

**Keywords:** C-reactive protein, T cells, monocytes, CD80/CD28 pathway, Belatacept, innate immunity

## Abstract

**Introduction:**

T cells are major components of the immune system. Their activation requires interaction between the T cell receptor and co-stimulatory molecules, crucial during infection, inflammation, and allogeneic rejection. Monomeric CRP (mCRP) is a known modulator of inflammation and particularly the innate immune response, however its interaction with T cells as part of the adaptive immune response remains unclear.

**Methods:**

Peripheral blood mononuclear cells (PBMC) and T cells were isolated. Flow cytometric analysis was conducted to evaluate Fcγ receptor CD16 expression on T cells, the binding of CRP to T cells, and its impact on proliferation and apoptosis. T cell activation was assessed after 1, 2, 3, 5 and 7 days by assessing CD69 and CD25 expression, and under various conditions including coculture with monocytes and several inhibitory factors.

**Results:**

T cells express CD16 that binds mCRP in a concentration-dependent manner, and particularly on activated T cells. While mCRP reduces apoptosis and accelerates proliferation in T cells, it does not independently activate them. However, activation of monocytes by mCRP leads to T cell activation, indicating a direct cell to cell interaction during CRP-induced activation. This effect could be alleviated by inhibition of the CD80/CD28 pathway.

**Conclusion:**

CRP does not activate T Cells directly but via PI3-kinase-dependent activation of monocytes and subsequent CD80/CD28 cell to cell contact. The findings suggest the effects of CRP on T cells depend on their environment and the presence of other proinflammatory agents.

## 1 Introduction

T cells constitute a pivotal component of the adaptive immune system. They can evolve in various subpopulations with distinct functions. Among others, T cells are involved in acute and chronic infections, inflammation, cancer, autoimmune disease, and alloreactivity (1).

T cells can be subdivided into cytotoxic T cells (CD8+) and T helper cells (CD4+). They further diversify into regulatory and non-regulatory T cells both consisting of naïve T cells, which are defined by expression of CD45RA (2), effector and memory T cells (3). T helper cells can differentiate into subpopulations depending on the surrounding cytokine milieu and the stimulation. The subtypes differ through expression of different surface markers and cytokine expression and can then be responsible for further activating other immune cells, such as B cells or macrophages (4, 5). TH1 cells (CD183+ CD196-) are essential for defense against intracellular pathogens and, like TH17–1 cells (CD183+ CD196+), produce IFN-γ. TH2 cells (CD183- CD196-) are involved in defense against extracellular parasites. TH17 cells (CD183- CD196+) act against extracellular bacteria and produce, among others, IL-17A (4, 6, 7).

Besides activation of the T cell receptor, productive T cell activation depends on co-stimulation of other cell surface receptors (8, 9). One notorious costimulatory molecule is CD28, often stimulated by CD80 expression on antigen-presenting cells, displaying the B7-CD28 pathway (10). Antigen-presenting cells like dendritic cells, but also monocytes or macrophages are often involved in co-stimulatory and co-inhibitory pathways (11–13). In the human organism, the interaction between T cells and antigen-presenting cells occurs during infections, inflammation, or rejection of allogeneic transplantations.

Another participator in the regulation of the immune system is acute phase reactant C-reactive protein (CRP), which is phylogenetically highly conserved and part of the innate immune system (14). Serum levels increase postoperatively, in ischemia-reperfusion injury, in tumors or during infections. CRP exists in various conformations and configurations in the organism, such as pentameric CRP (pCRP), activated pCRP (pCRP*), or monomeric CRP (mCRP) (15, 16). pCRP* and mCRP are attributed to strong proinflammatory properties, mainly via activation of the complement system (14, 17–19). Its interactions with immune cells occur through different ligands and receptors, such as Fcγ receptors (14, 18, 20) or the internalization of mCRP into the cell membrane (14). Previous studies have demonstrated that mCRP is able to activate monocytes (18), which enhances the production of proinflammatory cytokines, such as IL-1β, IL-6, and TNFα (21), and the production of reactive oxygen species via a complement-dependent manner (22). Monocytes circulate in the bloodstream, much like T cells, and are predominantly involved in the innate immune response (23, 24).

The interaction between pCRP and mCRP with monocytes, the complement system, and other parts of the immune system has been investigated frequently (21, 22, 25, 26). However, the impact of CRP and its isoforms on T cells has remained incompletely explored. This study investigates the direct influence of the two CRP isoforms pCRP and mCRP on the proliferation, apoptosis, and activation of T cells, and further explores their interaction with activated monocytes, providing crucial insights in inflammatory cell-cell interactions.

## 2 Methods

### 2.1 Preparation of pCRP and mCRP

Human pCRP was dialyzed in PBS over night at 4°C using a 0.5–3 ml dialysis cassette (Slide-A-Lyzer, Thermo Scientific, 10.000 MWCO). mCRP was generated by incubating pCRP for 1 h with 10 mM EDTA and 8 mM Urea at 37°C as previously described (27, 28). Dialyzing continued in Tris-HCl buffer at 4°C, and mCRP was harvested and reduced in 10 mM DTT buffer for 90 min at 37°C under constant stirring. The solution was again dialyzed in Tris-HCl buffer overnight at 4°C. The reduced and dialyzed mCRP was harvested under sterile conditions and then measured using QuBit Fluorometer (Thermo Fisher Scientific, Waltham, Massachusetts, USA), according to manufacturer’s instructions.

For binding assays, pCRP and mCRP were dialyzed 1:500 in PBS pH 8.3 overnight at 4°C before being conjugated to Texas Red (TR) maleimide (483 AAT Bioquest, Sunnyvale, California, USA). The TR-CRP conjugation reaction was run for 1 h at 37°C using a 10:1 molar ratio, then dialyzed overnight at room temperature 1:500 in PBS pH 7.2-7.4. Binding assays were analyzed after 24h.

### 2.2 Isolation of PBMC, T cells, and monocytes

Whole blood was taken from healthy voluntary blood donors under standardized conditions and processed within 30 min. PBMC were separated from whole blood via density gradient centrifugation as previously described (21). T cells were magnetically isolated using EasySep™ Human T Cell Isolation Kit (STEMCELL Technologies, Cologne, Germany, #18000) according to manufacturer’s instructions. Monocytes were isolated with Magnetic Beads Monocyte Isolation Kit CD14+ without CD16+ depletion (STEMCELL Technologies, Cologne, Germany, #19058) according to manufacturer’s instructions.

### 2.3 Cell cultures

Human PBMC and T cells were incubated in RPMI cell culture medium with 10% FBS and 1% P/S either alone or with Dynabeads™ Human T-Activator CD3/CD28 (1:1 (bead number:cells), Gibco by Thermo Fisher Scientific, Waltham, Massachusetts, USA) for 6 h, 24 h, 48 h and 3, 5 or 7 days or with addition of 25 µg/ml, 50 µg/ml or 100 µg/ml mCRP and 25 µg/ml, 50 µg/ml or 100 µg/ml pCRP, respectively. Cells were seeded at 10^6^ cells/ml. Experiments regarding the expression of CD16 were conducted after 24 h of stimulation with CD3/CD28 Dynabeads. Proliferation assays were incubated with 1:5000 CFSE CellTrace (Invitrogen™ C34554, Thermo Fisher Scientific, Waltham, USA) before culture and stimulated with CD3/CD28 Dynabeads as described above, then analyzed after 5 days. The same conditions were applied for apoptosis assays regarding the expression of Annexin V. Cocultures were incubated with T cells and Monocytes in equal parts in OpTmizer cell culture medium (Thermo Fisher Scientific, Waltham, Massachusetts, USA) with 1% P/S in 96-well plates. The transwell cultures were incubated for 3 days using inserts with pore size 0.4 µm. When Belatacept was used, 10 µg/ml were added to the cell cultures before starting the incubation. Wortmannin, a PI3-Kinase inhibitor, which is able to inhibit the influence of mCRP on monocyte activation (26), was used at 25 µg/ml and with 10 min incubation at 4°C. CD80 expression on monocytes was assessed after 15 min of stimulation as previously described (21).

### 2.4 Antibodies and staining

To assess activation parameters cell suspensions with T cells were stained with anti-CD3 (1:200, BW264/56, VioBlue, Miltenyi Biotec, Bergisch Gladbach, Germany, RRID: AB_10831672), anti-CD8 (1:200, REA734, FITC, Miltenyi Biotec, Bergisch Gladbach, Germany, RRID: AB_2659233), anti-CD4 (1:100, SK3, BV650, BD Biosciences, Franklin Lakes, New Jersey, USA, RRID: AB_2744425), anti-CD183 (1:20, G025H7, BV605, BioLegend, San Diego, California, USA, RRID: AB_2563157), anti-CD196 (1:100, G034E3, PE-Cy7, BioLegend, San Diego, California, USA, RRID: AB_10916518), anti-CD69 (1:33, FN50, BV785, BioLegend, San Diego, California, USA, RRID: AB_2561370) and anti-CD25 (1:20, 2A3, APC-R700, BD Biosciences, Franklin Lakes, New Jersey, USA, RRID: AB_2744339). CRP-Binding was evaluated by adding Texas Red-conjugated CRP, as described above. Expression of FcγR subtypes was analyzed by anti-CD16 (1:100, 3G8, PE-Cy7, BD Pharmingen, Franklin Lakes, New Jersey, USA, RRID: AB_396850), anti-CD32 (1:20, IV.3, FITC, Stem Cell Technologies, Vancouver, Canada, RRID: AB_519586) and anti-CD64 (1:50, 10.1, FITC, Thermo Fisher Scientific, Waltham, Massachusetts, USA, RRID: AB_2536511). IgG1 K PE-Cy7 (1:100, BD Pharmingen, Franklin Lakes, New Jersey, USA, RRID: AB_396914) served as isotype control for anti-CD16-PE-Cy7. To assess costimulation with CD3/CD28 beads and cytokine production over time anti-CD4 (1:20, RPA-T4, eFluor 450, Thermo Fisher Scientific, Waltham, Massachusetts, USA, RRID: AB_1272057), anti-CD8 (1:50, RPA-T8, BV711, BD Biosciences, Franklin Lakes, New Jersey, USA, RRID: AB_2744463), anti-CD45RA (1:20, HI100, BV510, BioLegend, San Diego, California, USA, RRID: AB_2561947), anti-CD25 (1:20, 2A3, APC-R700, BD Biosciences, Franklin Lakes, New Jersey, USA, RRID: AB_2744339), anti-CD69 (1:20, FN50, BV786, BD Biosciences, Franklin Lakes, New Jersey, USA, RRID: AB_2738441), anti-IFN-γ (1:50, REA600, PE, Miltenyi Biotec, Bergisch Gladbach, Germany, RRID: AB_2733717), anti-TNF-α (1:50, Mab11, FITC, Thermo Fisher Scientific, Waltham, Massachusetts, USA, RRID: AB_465423), and anti-IL-17A (1:50, CZ8-23G1, APC, Miltenyi Biotec, Bergisch Gladbach, Germany, RRID: AB_2752081) were used. Cytokines were stained after permeabilizing the cells with BD Cytofix/Cytoperm™ (BD, Franklin Lakes, New Jersey, USA).

To assess apoptosis, PBMC were stained with anti-CD8 (1:100, SK1, APC-Cy7, BioLegend, San Diego, California, USA, RRID: AB_2044006), anti-CD4 (1:100, RPA-T4, eFluor450, Thermo Fisher Scientific, Waltham, Massachusetts, USA, RRID: AB_1272057), and anti-Annexin-V (1:20, FITC, BD Biosciences, Franklin Lakes, New Jersey, USA, RRID: AB_2665412) after 5 days of incubation. The same antibodies were used for proliferation assays.

Monocytes were stained with anti-HLA-DR (1:50, TU36, FITC, BD Pharmingen, Franklin Lakes, New Jersey, USA, RRID: AB_395942), anti-CD14 (1:50, M5E2, Pacific Blue, BD Biosciences, Franklin Lakes, New Jersey, USA, AB_397041), anti-CD16 (1:50, 3G8, PE-Cy7, BD Pharmingen, Franklin Lakes, New Jersey, USA, RRID: AB_396850) and anti-CD80 (1:50, REA661, APC, Miltenyi Biotec, Bergisch Gladbach, Germany, RRID: AB_2751432), as well as anti-CD2 (1:50, RPA-2.10, PE, BD, Franklin Lakes, New Jersey, USA, RRID: AB_395734), anti-CD19 (1:50, HIB19, PE, BD, Franklin Lakes, New Jersey, USA, RRID: AB_10893795), anti-CD15 (1:50, VIMC6, PE, Miltenyi Biotec, Bergisch Gladbach, Germany, RRID: AB_871623), anti-CD56 (1:50, MY31, PE, BD, Franklin Lakes, New Jersey, USA, RRID: AB_2868831), and anti-NKp46 (1:50, BAB281, PE, BD, Franklin Lakes, New Jersey, USA, RRID: AB_396974) for negative lineage as previously described (21).

### 2.5 Flow cytometric analysis

Flow cytometric analysis was performed immediately after staining using LSR Fortessa™ (BD Biosciences, Franklin Lakes, New Jersey, USA) with standard filter and mirror configuration. Data was acquired by BD FACSDiva Software and further evaluated using FlowJo Software (Version 10.7.1., BD, Franklin Lakes, New Jersey, USA). T cells were visualized based on size and granularity (FSC/SSC), as well as doublets and dead cell exclusion. Then, T cell subsets were further specified into CD4+ T helper cells and CD8+ cytotoxic cells. CD4+ helper cells were subdivided into Th1 (CD183+ CD196-), Th2 (CD183- CD196-), Th17 (CD183- CD196+) and Th17-1 (CD183+ CD196+) based on the expression of CD183 and CD196 (**Supplementary Figure 1**). Notably, CD183 and CD196 expression were evaluated exclusively on CD4+ T cells. To quantify T cell activation, CD25 and CD69 expression was measured and corrected for fluorescence minus one (FMO) controls respectively. Intracellular cytokines TNFα, IFNγ, and IL17-A were assessed according to the protocol described above.

### 2.6 Statistical analysis

Statistical analysis was performed with GraphPad Prism Software, Version 10.4.1. All data was tested for normality with Shapiro-Wilk test. For comparing more than two groups, either one-way ANOVA or Friedman test followed by Tukey’s or Dunn’s multiple comparisons test was conducted. For comparing two groups, two-tailed (ratio) paired t-test or Wilcoxon matched-pairs signed-rank test was used. P values < 0.05 were considered significant.

## 3 Results

### 3.1 T cells express CD16 in an activation dependent manner

**Figure 1:**
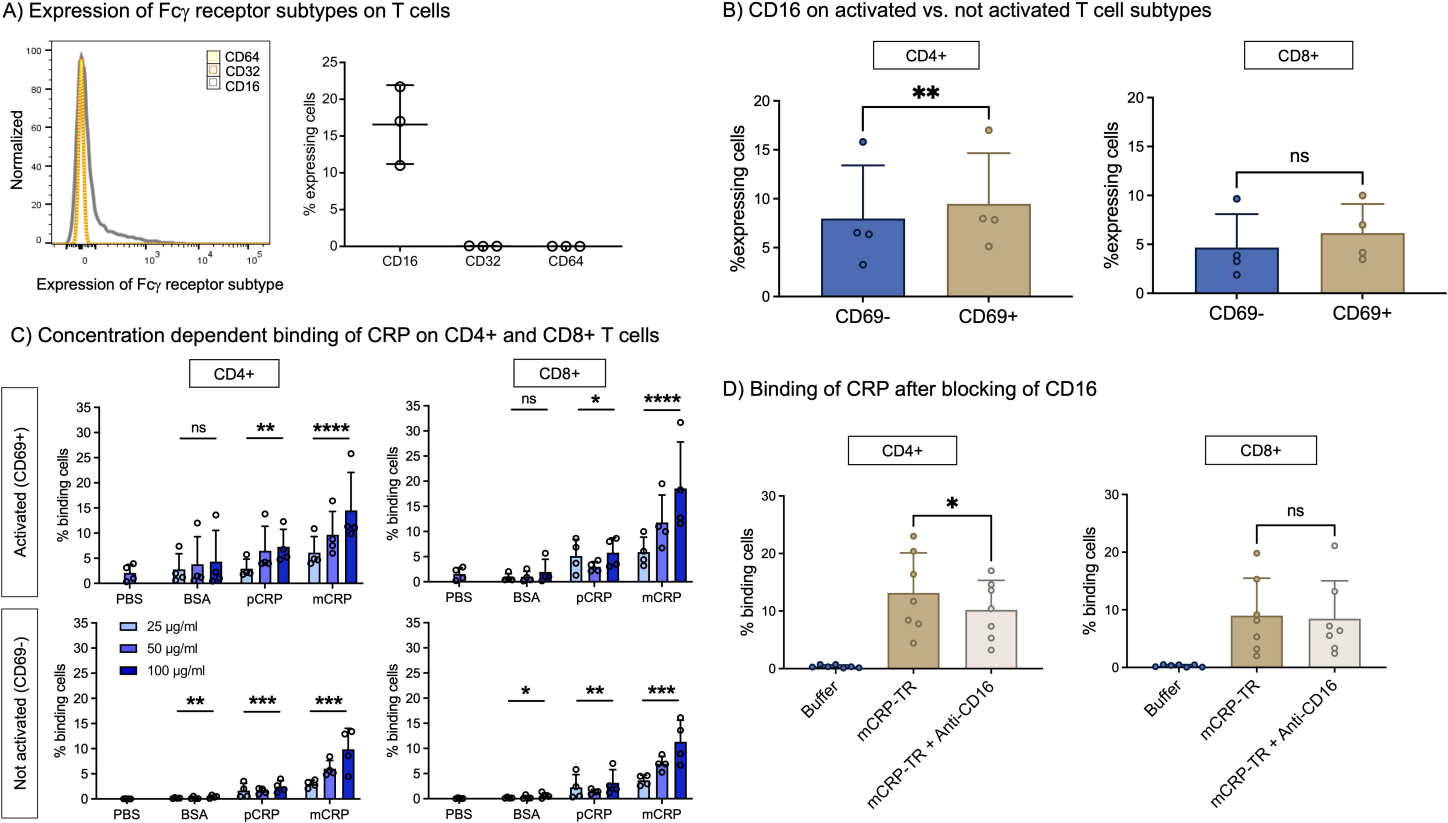
**(A)** Expression of Fcγ receptor subtypes on T cells. Freshly taken whole blood was stained with antibodies against Fcγ receptor subtypes (CD16, CD32, CD64) and assessed in flow cytometry. CD16 is expressed by T cells, CD32 and CD64 not. n=3. **(B)** CD16 on activated vs. not activated T cell subtypes. PBMC were incubated with 12.5 µl/ml CD3/CD28 beads (1:2 bead number:cells) at 37°C and 5% CO_2_ for 24 h and additionally stained with antibodies against CD4, CD8 and CD69 to assess subtype and activation state. CD16 is significantly more often expressed by activated (CD69+) than by non-activated T cells (CD69-) in CD4+ T cells. n=4. Paired t-test. **(C)** Concentration-dependent binding of CRP isoforms on CD4+ and CD8+ T cells. PBMC were incubated with 12.5 µl/ml CD3/CD28 (1:2 bead number:cells) beads at 37°C and 5% CO_2_ for 24 h Binding of BSA, pCRP and mCRP at 25 µg/ml, 50 µg/ml and 100 µg/ml was assessed by conjugation with PE-Texas Red. Brakets indicate results of Friedman’s test in comparison to PBS. mCRP and pCRP bind significantly to CD4+ and CD8+ T cells in comparison to control. n=4. **(D)** Binding of mCRP after blocking of CD16. PBMC were incubated with 12.5 µl/ml CD3/CD28 beads (1:2 bead number:cells) at 37°C and 5% CO_2_ for 24 h Before adding fluorophore-conjugated mCRP, cells were incubated with anti-CD16 for 15 min. Blocking of CD16 significantly reduced mCRP binding on CD4+ T cells. Results shown in mean and standard deviation. n=7. *p<0.05, **p<0.01, ***p<0.001, ****p<0.0001, ns = not significant.

CRP exerts its effects on cells including but not being limited to via Fcγ receptors I, II, and III (26, 29). Fcγ receptor I is also known as CD64, FcγRII as CD32, and FcγRIII as CD16. On average 16.5% of all T cells express CD16 on their surface (**Figure 1A**). CD32 and CD64 are not detected on T cells.

To further examine the expression of CD16, PBMC from healthy donors were stimulated with CD3/CD28 beads and additionally stained with antibodies against CD4, CD8, and CD69 to assess co-expression on activated versus non-activated CD4+ versus CD8+ T cells. **Figure 1B** shows that activated CD4+ T cells express significantly more CD16 than those not activated (9.5% vs. 8.0%, p<0.01, n=4). In contrast, we could not detect a significant difference in CD16 expression between activated or not activated CD8 T cells (6.2% vs. 4.7%, p>0.05, n=4).

### 3.2 mCRP binds to T cells in a concentration- and activation-dependent manner via CD16

Given the fact that we could detect the expression of the CD16 on T-cells we subsequently aimed to prove whether T cells are also able to bind mCRP and pCRP.

mCRP binding to CD4+ and to CD8+ T cells is dependent on its concentration. On activated T cells (CD69+), after 24h, the binding capacity of mCRP is higher than on non-activated cells. At 100 µg/ml almost 15% of activated CD4+ and 18.5% of activated CD8+ cells are binding mCRP (p<0.0001, n=4). In addition, pCRP is also able to bind to T cells, but to a lesser degree (**Figure 1C**).

As proof of concept, the binding of mCRP to T cells via FcγRIII was inhibited by blocking CD16. The preincubation of cells with anti-CD16 3G8 and subsequent incubation with Texas Red-conjugated mCRP (mCRP-TR) resulted in significantly less binding of mCRP-TR to CD4+ cells (13.17% vs. 10.22%, p<0.05, n=7) (**Figure 1D**).

### 3.3 mCRP reduces apoptosis and accelerates proliferation in T cells

Based on our results shown above, we tested the hypothesis that mCRP is able to influence the functionality of T cells.

**Figure 2:**
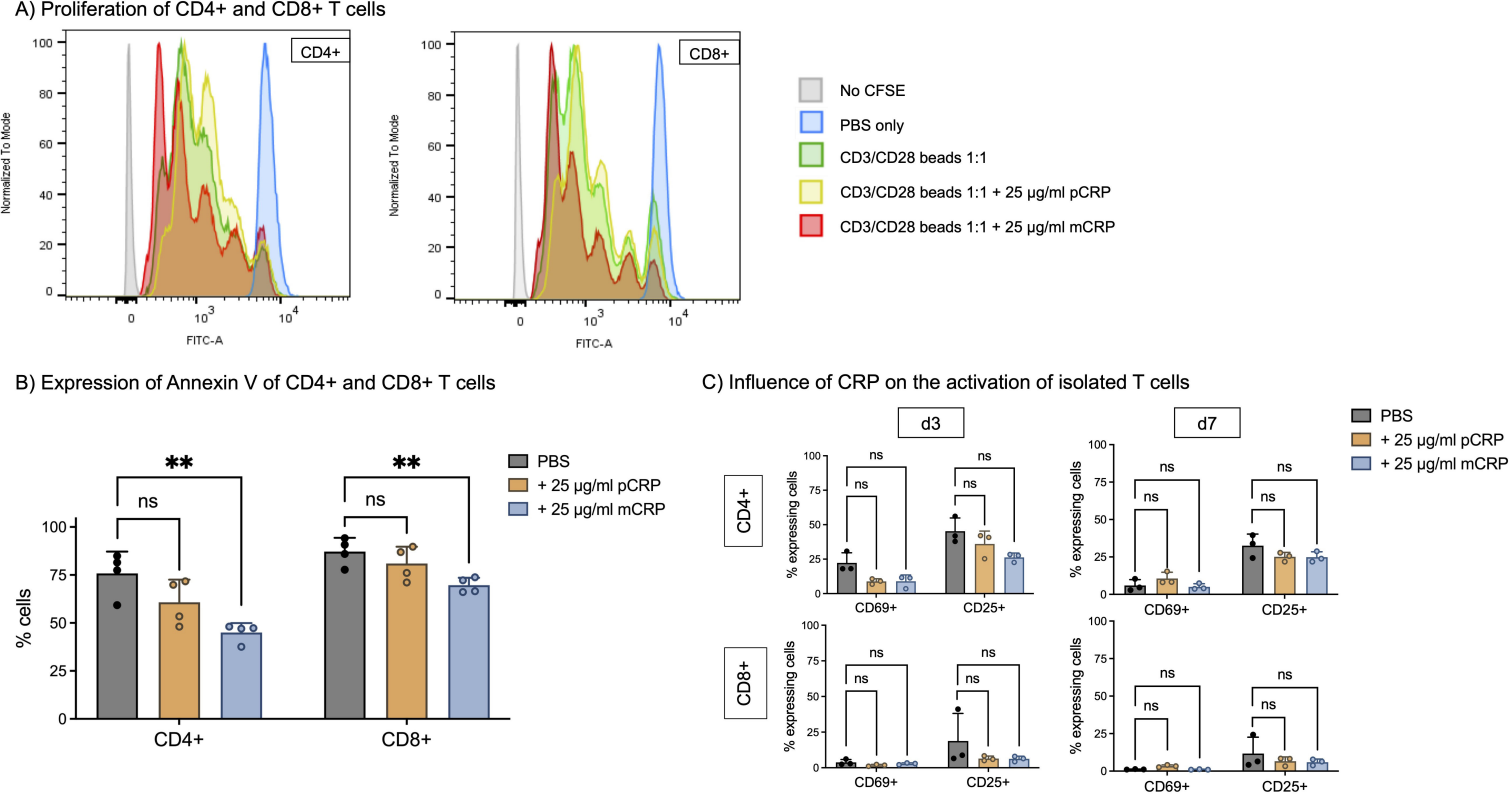
**(A)** Proliferation of CD4+ and CD8+ T cells. PBMC were incubated for 5 days at 37°C and 5% CO_2_ with 12.5 µl/ml CD3/CD28 beads (1:2 bead number:cells). T cell proliferation was assessed with CFSE (CellTrace) to track following generations. 25 µg/ml pCRP and mCRP were added respectively. In the control group (PBS), no T cell proliferation was observed. Flow cytometry analysis revealed distinct peaks, each corresponding to a successive generation of dividing T cells and thereby reduced CFSE-fluorescence, with younger generations located towards the left of the x-axis. Stimulation with mCRP significantly enhanced the proliferation of both CD4+ and CD8+ T cells in comparison to CD3/CD28 beads, whereas pCRP did not have this effect. **(B)** Apoptosis of CD4+ and CD8+ T cells. PBMC were incubated for 5 days at 37°C and 5% CO_2_. 25 µg/ml pCRP and mCRP were added. Apoptosis was assessed by Annexin V staining. mCRP decreases the percentage of apoptotic cells in comparison to PBS by 30% (CD4+) and by 17% (CD8+). n=4. **(C)** Influence of CRP isoforms on the activation of isolated T cells. T cells were isolated via magnetic sorting and incubated with PBS, 25 µg/ml pCRP, and 25 µg/ml mCRP for 3 and 7 days. There was no significant influence on the percentage of activated cells with early activation marker CD69+ nor late activation marker CD25+. Results are shown in mean and standard deviation. n=3. **p<0.01. ns = not significant.

We used CFSE cell tracking to investigate T cell proliferation. Supplemental mCRP accelerates cell proliferation in comparison to CD3/CD28 beads alone and pCRP, indicated by a decreased first generation and increased youngest population in CD4+ as well as in CD8+ T cells, whereas the control population stimulated with solvent control (PBS) did not show any proliferation. Interestingly, pCRP had no significant effect on cell proliferation capacity (**Figure 2A**).

Accordingly, mCRP significantly reduces apoptosis in T cells compared to PBS only. Cells were prepared according to the proliferation assay protocol and apoptosis was assessed by staining for Annexin V binding. For CD4+ T cells, incubating with mCRP significantly reduced the rate of apoptotic cells from >75% to 45% (p<0.01, n=4). In CD8+ T cells, the rate significantly decreased from >87% to <70% by addition of mCRP (p<0.01, n=4). Again, pCRP had no statistically significant effect on CD4+ T cells or CD8+ cells (p>0.05, n=4) (**Figure 2B**).

Meanwhile, T cell subtype distribution is not influenced by addition of pCRP nor mCRP. The proportion of CD4+, TH1, TH2, TH17, TH17–1 and CD8+ T cells did not differ significantly after incubation with pCRP or mCRP (p>0.05, n=3) (**Supplementary Figure 2**).

### 3.4 mCRP alone does not activate T cells

Since we were able to show the binding of CRP on T cells and its impact on proliferation and apoptosis, we next investigated its influence on T cells’ activation state. The gating strategy is shown in **Supplementary Figure 3**.

There was no significant activation of T cells after incubation with pCRP or mCRP (**Figure 2C**). After 3 days, 22.3% of unsupplemented CD4+ T cells express CD69 and 45.3% CD25, whereas after 3 days of incubation with mCRP those amounts are approximately 8.9% and 22.6%, respectively (p>0.05, n=3). For CD8+ T cells, similar tendencies are observed (CD69+ PBS 3.8% vs. mCRP 2.8%, CD25+ PBS 18.8% vs. mCRP 6.3%, p>0.05, n=3), although the overall activation is lower.

### 3.5 mCRP reduces activation in pre-stimulated T cells

As CRP stimulation was insufficient to modulate T cell activation we next investigated the effect of CRP stimulation with simultaneous costimulation.

CD3/CD28 beads are commonly used to stimulate T cells (30, 31) and were used as a baseline stimulation 1:1 (bead number:cells), 50 µg/ml pCRP and mCRP were added respectively. **Supplementary Figure 4** and **Supplementary Figure 5A** show representative gating strategies to evaluate T cell activation via CD69 and CD25 expression and cytokine production of TNFα, IFNγ and IL17-A.

For CD3/CD28 beads only, the number of CD69 expressing cells increased during the first 24 h and decreased after 48 h for CD4+ T cells, but not for CD8+ T cells. In both cases, CD25+ T cells increased until day 5.

**Figure 3:**
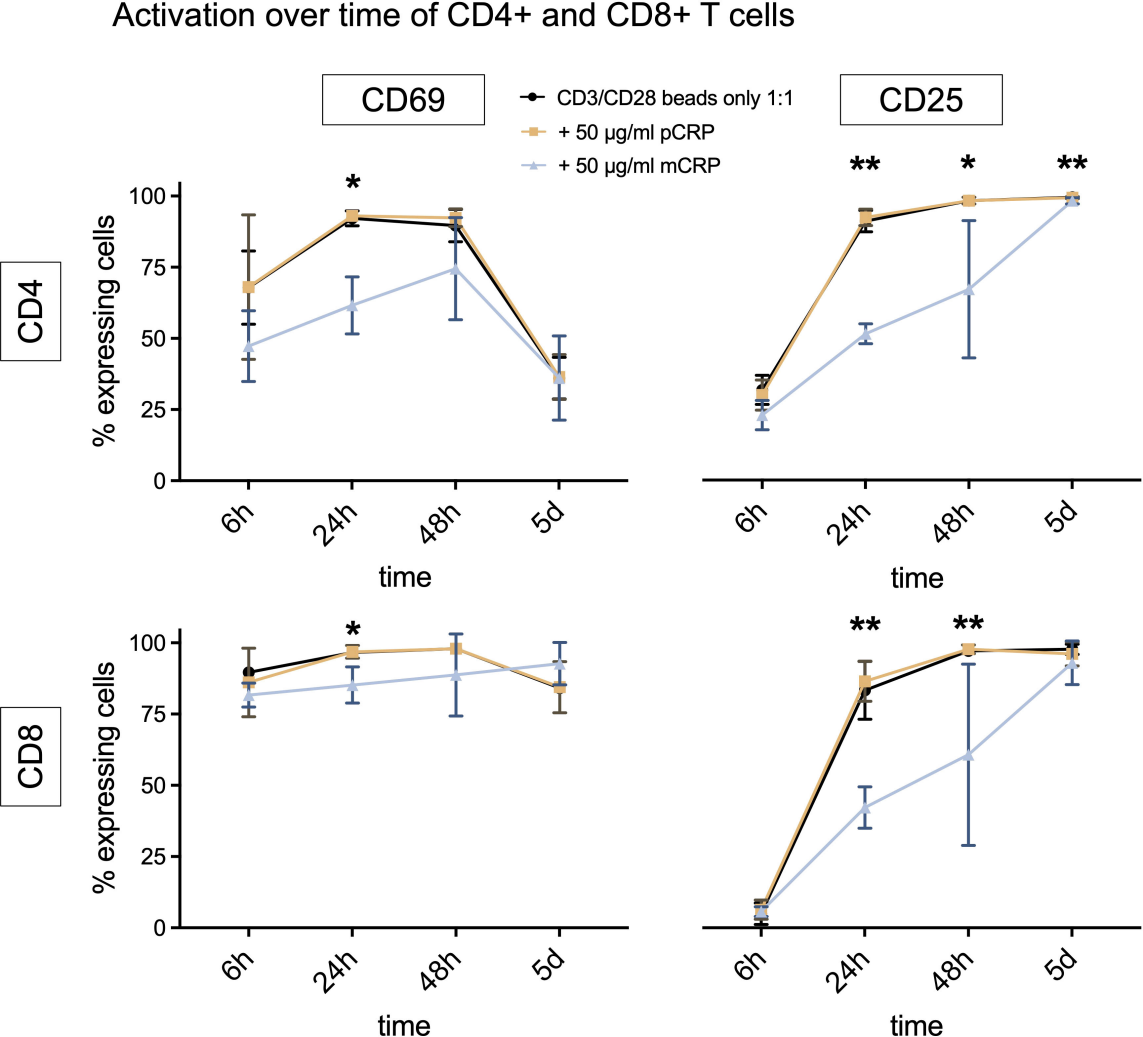
Activation over time of CD4+ and CD8+ T cells, costimulation with CD3/CD28 beads. The curve displays a time course of activation markers CD69+ and CD25+ for CD4+ and CD8+ T cells when stimulated with CD3/CD28 beads only or additionally 50 µg/ml pCRP or mCRP. All results are displayed as means and standard deviation. n=5. *p<0.05, **p<0.01.

The pCRP group shows a similar activation pattern to the control group with CD3/CD28 beads only (**Figure 3**). At every time point (except for CD8+ T cells on day 5), the activation of T cells, which were incubated with mCRP, lagged behind. There were statistically significant differences between the groups after 24 h for CD4+ as well as CD8+ T cells in terms of CD69 and CD25 expression (CD69 p<0,05, CD25 p<0.01, n=5). After 48 h, there is still a statistically significant difference between the groups regarding the expression of CD25 in CD4+ (p<0.05, n=5) as well as in CD8+ T cells (p<0.01, n=5). After 5 days, only CD4+ T cells incubated with mCRP still expressed less CD25+ than the group with CD3/CD28 beads only (p<0.01, n=5).

Neither pCRP nor mCRP appear to have a consistent impact on cytokine production (**Supplementary Figure 5B**). The production of IL-17A was generally very low but showed statistically significant differences between the groups for CD4+ and CD8+ T cells (p<0.05, n=5).

### 3.6 mCRP activated monocytes activate T cells in coculture

Costimulation with CD3/CD28 beads did not enhance mCRP mediated T cell activation. Since monocytes are known to be activated by CRP (21), we next aimed to characterize monocyte dependent effects of CRP. As pCRP did not show any effect on T cells, following we focused on the effects of mCRP. We have previously shown that mCRP but not pCRP, activates monocytes (32). Therefore, we focused on the specific role of mCRP on monocyte-mediated T cell activation. Also, we constrained on assessing CD69 after 3 days to determine T cell activation.

**Figure 4:**
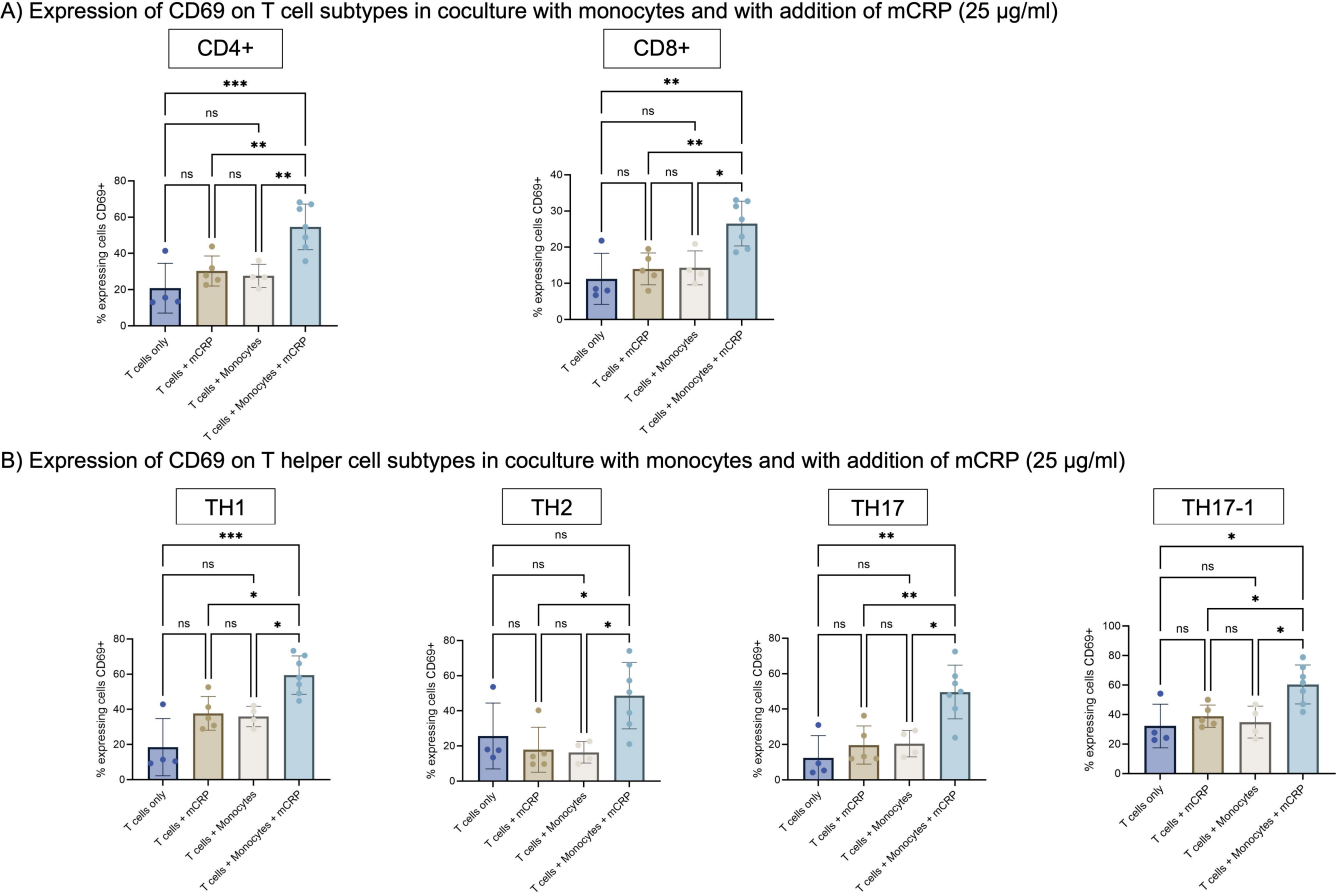
**(A)** Expression of CD69 on T cell subtypes in coculture with monocytes and with addition of mCRP. T cells were stimulated with 25 µg/ml mCRP either alone or in coculture with monocytes. T cells only served as an additional control group. Activation was assessed after 3 days via expression of surface marker CD69. The addition of mCRP to coculture with monocytes lead to a significant higher expression of CD69 on T cells in comparison to T cells only, monocytes or mCRP alone. n=7. **(B)** Expression of CD69 on T helper cell subtypes in coculture with monocytes and with addition of mCRP. As for CD4+ T helper cells, the addition of mCRP to coculture with monocytes lead to a significant higher expression of CD69 on T helper cell subtypes. Results are shown in mean and standard deviation. n=7. *p<0.05, **p<0.01, ***p<0.001. ns = not significant.

Only a small number of T cells express CD69 in absence and presence of mCRP (**Figure 4A**). In coculture with monocytes, the number of CD69 expressing cells remained similar for CD4+ and CD8+ T cells, as well as all helper cell subtypes (**Figure 4B**).

Only when T cells were cultured with monocytes and additionally with mCRP, the number of activated cells expanded significantly in comparison to both, mCRP only and monocytes only. Among CD4+ T cells, the amount of activated CD69+ cells increased significantly to 54.6% (p<0.001, n=7). For CD8+ T cells, the amount of CD69 expressing cells advanced significantly to 23.2% (p<0.01, n=7). The same effect applies to all helper cell subtypes.

In contrast, the transwell culture of monocytes and T cells did not show any significant changes after adding mCRP or pCRP, neither for CD69 nor CD25 (p>0.05, n=4) (**Supplementary Figure 6**).

### 3.7 mCRP increases CD80 expression on monocytes via a PI3-kinase-dependent mechanism. Activation of T cells via mCRP-stimulated monocytes is dependent on CD80-CD28-interaction

Since our previous experiments have indicated that a direct cell contact is necessary to activate T cells via mCRP-stimulated monocytes, we aimed to elucidate the mechanism of monocytes and T cells interaction leading to mCRP-induced activation of T cells. We hypothesized that this interaction might involve CD80 on monocytes and CD28 on T cells, which is a common pathway of interaction between antigen-presenting cells and T cells (11).

Freshly taken whole blood was incubated with mCRP and Wortmannin, a PI3-kinase inhibitor. Then, monocytes were analyzed in flow cytometry as described previously (21) and stained with anti-CD80-APC to assess their activation.

**Figure 5:**
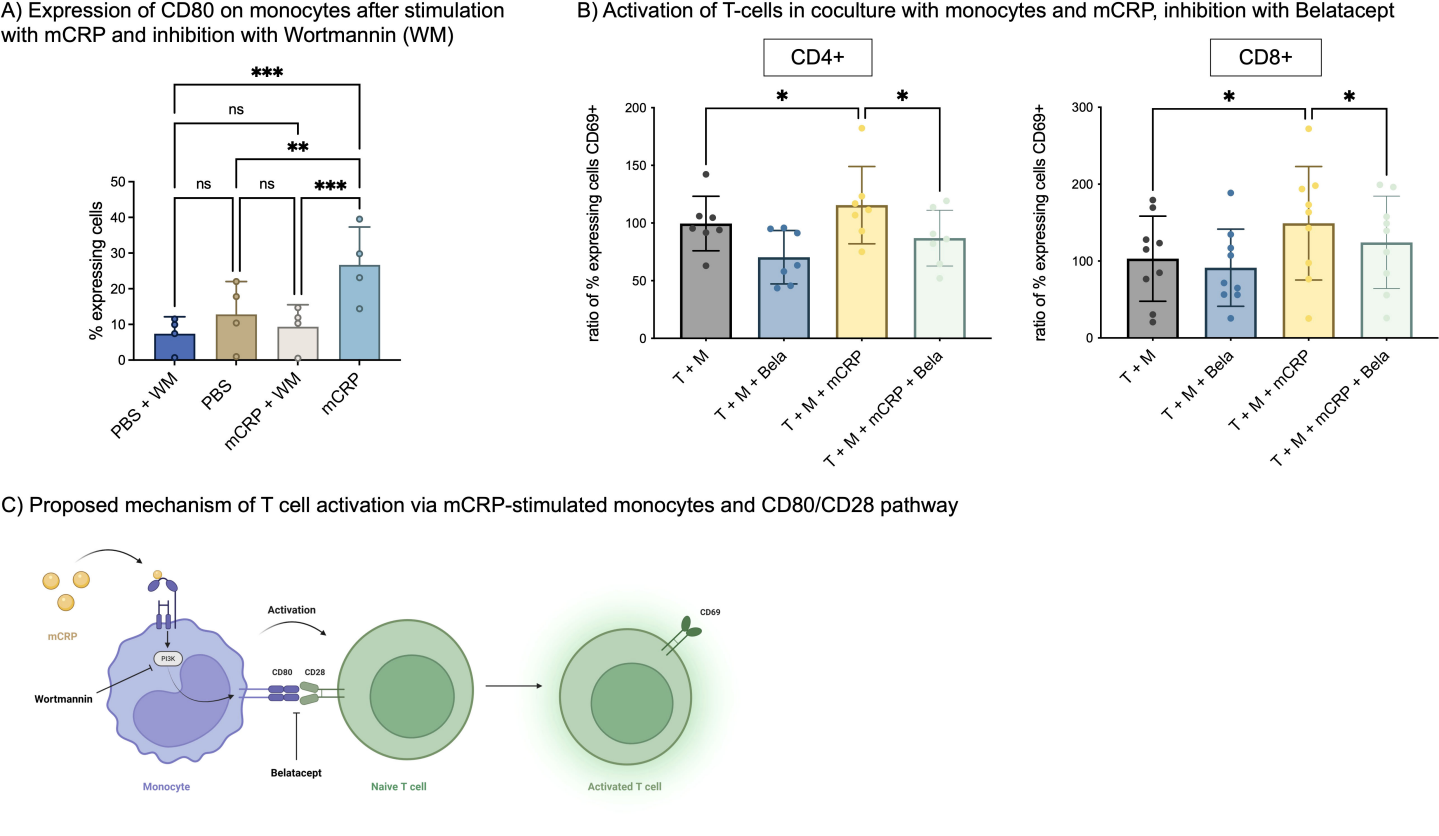
**(A)** Expression of CD80 on monocytes. Freshly taken whole blood was incubated with 50 µg/ml mCRP and 25 µg/ml Wortmannin (WM), a PI3-kinase inhibitor. Monocytes were identified as described previously (21) and stained with anti-CD80-APC. n=4. **p<0.01, ***p<0.001. ns = not significant. **(B)** Activation of T-cells in coculture with monocytes and mCRP, inhibition with Belatacept. A coculture of T-cells and monocytes was stimulated with 50 µg/ml mCRP. For inhibition, 10 µg/ml Belatacept was added and incubated for 3 days. T-cells were stained with anti-CD69 to assess activation. Data was normalized on T+M control group. n=9. *p<0.05. **(C)** Proposed mechanism of T cell activation via mCRP-stimulated monocytes and CD80/CD28 pathway. Monocytes are activated by mCRP via a PI3-kinase-dependent mechanism, which increases their expression of CD80. CD80 interacts with T-cells’ CD28, thereby increasing T cell activation in terms of CD69 expression. This interaction can be inhibited by Belatacept.

After incubation with mCRP, the number of CD80 expressing monocytes increased significantly from 12.8% to 26.7% (p<0.01, n=4). Prior incubation with PI3-kinase inhibitor Wortmannin decreased this activation again significantly to 9.4% (p<0.001, n=4) (**Figure 5A**).

In a second step, T-cells were incubated in coculture with monocytes and mCRP as described above, and additionally inhibited with 10 µg/ml Belatacept, a fusion protein linking to CTLA4 (CD28) and thereby blocking T cell costimulation.

For CD4+ T cells, stimulation with mCRP again increased the ratio of CD69+ T cells to 115.5% (p<0.05, n=9) compared to unstimulated coculture. Simultaneous addition of Belatacept, decreased CD69+ T cells to 86.91% (p<0.05, n=9). A similar effect was observed with CD8+ T cells, where mCRP induced 149.2% (p<0.05, n=9) activated T cells and Belatacept significantly decreased this activation to 124.3% (p<0.05, n=9) **Figure 5B**.

An overview of the proposed mechanism behind mCRP-stimulated monocyte-dependent T cell activation is shown in **Figure 5C**.

## 4 Discussion

Here, we describe the interaction between CRP isoforms pCRP and mCRP and T cells. T cells express CD16, especially when they are activated. mCRP is able to bind on T cells, depending on CD16, and induces proliferation. mCRP did not increase expression of activation parameters CD69 and CD25 on T cells and was able to reduce activation significantly when co-stimulated with CD3/CD28 beads. Only in coculture but not in transwell-culture with monocytes mCRP did have a stimulatory effect on T cells, suggesting the involvement of a direct cell to cell interaction. We assume a PI3-kinase dependent upregulation of CD80 on monocytes after stimulation with mCRP, leading to activation of T cells via CD80-CD28 interaction. This was confirmed by successfully blocking the mCRP-mediated monocyte-dependent activation of T cells with Belatacept, an immunosuppressor binding to CD80. Although pCRP was able to bind on T cells as well, it had no further effect on T cells, as observed for monocytes before (21).

The binding of mCRP on T cells is concentration-dependent and especially occurred when they were activated, correlating with their higher expression of CD16. Although the observed difference may appear modest, the significantly higher expression of CD16 on activated CD4+ T cells indicates an enhanced responsiveness to mCRP upon activation, thus amplifying the proinflammatory effects of mCRP. Anyway, blocking CD16 did not lead to a completely inhibited binding, indicating other involved mechanisms between mCRP and T cells next to CD16. On granulocytes and monocytes, CD32 and CD64 are described to be receptors for CRP (33, 34). Since we did not detect their expression on T cells, unspecific binding or signal transduction via internalization of mCRP provide an alternative (14). This also refers to CD8+ T cells, where we could not see a significant reduction of mCRP binding after blocking CD16. Even though CD32 and CD64 were not detected on T cells in our experiments, low-level or inducible expression, particularly of high-affinity FcγRI, could still contribute to mCRP binding (35–38). Beyond FcγRs, other receptors, such as FcαRI (CD89), which is known to cross-react with CRP under inflammatory conditions, may also be involved (39). Scavenger receptors such as LOX-1 and CD36, which are involved in lipid uptake and are upregulated during inflammation, are further candidates for mCRP binding (40, 41). Finally, mCRP can interact with cell membranes and might bind receptor-independent via internalization or directly to lipid rafts. This mechanism is also known to initiate signaling pathways (26, 42, 43).

The binding of mCRP on T cells results in increased proliferation and decreased apoptosis of CD4+ as well as CD8+ T cells, as previously described for neutrophil granulocytes (20). Though, we did not observe any activation of isolated T cells when stimulated with mCRP. Under conditions of costimulation with CD3/CD28 beads, even a counterintuitive lower activation is observed in the presence of the otherwise proinflammatory mCRP. Only in coculture with monocytes the proinflammatory properties of mCRP do manifest in T cells. The observation that proliferation was nevertheless enhanced and apoptosis was inhibited may be due to the fact that these experiments were not performed with PBMC rather than isolated T cells. They also contain a population of monocytes (being the next most abundant after lymphocytes) that may contribute in part to the observed effects via the subsequently described mechanism.

These observations add a layer of complexity to the understanding of mCRP’s role in T cell regulation. The current literature on this topic is quite controversial.

In 1986, CRP was described to inhibit T cell autoactivation and suppress T cell proliferation (44). In contrast, Zhou et al. recently reported enhancement of T cell receptor signaling-dependent bystander activation of CD4+ T cells by mCRP (45), clearly showing the proinflammatory characteristics of the protein.

On the other hand, activated T cells are able to stimulate monocytes themselves in producing proinflammatory cytokines like TNF-α (46). Also, the production of IL-1β in monocytes depends on direct contact with stimulated T cells (47), next to stimulation via mCRP (21). Hence, the mCRP-triggered cell-cell contacts might not be one-way here. Furthermore, similar observations regarding the involvement of other leukocyte subtypes in T cell activation via CRP have previously been reported for dendritic cells (48, 49).

Our results indicate that although pCRP and mCRP are able to bind on T cells and mCRP increases their proliferation, neither pCRP nor mCRP significantly increased T cells in their activation state. Interestingly, when in coculture with monocytes mCRP unfolds its proinflammatory properties not only on monocytes (18, 21, 22), but passes them on T cells as well. Most likely, since mCRP increases the amount of CD80 expressing monocytes, as shown in our results, the activation cascade occurs, at least in parts, via the CD80/CD28 co-stimulation pathway. This hypothesis was further proven by their successful blockade with Belatacept and the subsequent reduced activation of T cells. Indeed, Belatacept is not only described as an antirheumatic drug, but has been used to suppress allograft reactivity *in vitro* (50) and *in vivo* (51). It inhibits costimulation via the CD80/CD28 pathway and can thereby suppress alloreactive CD4+ and CD8+ T cells, especially when used in combination therapy (50). The CD80/CD28 pathway forms a link between innate and adaptive immune responses (52). This may be of particular interest in preventing chronic rejection by helping to suppress the specific contribution of the innate immune system. This includes not only the response of antigen-presenting cells, such as dendritic cells or monocytes, but also the influence of CRP. mCRP, a well-known mediator of allograft rejection (32), therefore exhibits its effects not directly on T cells but via a monocyte-dependent activation cascade including the CD80/CD28 pathway. These observations suggest that, although mCRP alone does not directly activate purified T cells, signals from monocytes, particularly the upregulation of CD80, activate PBMC by providing the necessary co-stimulatory context for T cell activation via the CD80/CD28 pathway. In the clinical context of transplantation, heightened CD80 expression could disrupt tolerance mechanisms by facilitating stronger T cell responses against the allograft and thereby increasing the risk of rejection. This mechanism is supported by the successful blockade of the CD80/CD28 interaction with Belatacept and the subsequent reduced activation of T cells.

We focused on the CD80/CD28 pathway because it is a critical axis for T-cell activation and function, and its modulation by monocytes is likely one key mechanism in shaping immune responses in inflammation and immunity (53). Nonetheless, other pathways play a crucial role in T cell function and may be involved in T cell activation through mCRP and monocytes, such as PDL1/PD1, ICOSL/ICOS or TIM4/TIM1 pathway (11). Furthermore, CD80 is not only expressed on monocytes, but also by multiple other cell types, including B cells and dendritic cells (54). Having established that mCRP triggers T cell activation via CD80 on monocytes, further studies are necessary to investigate the involvement of additional possible mechanisms.

Transferring our findings into a clinical setting, T cells and monocyte interaction are involved in several disorders. The impact of mCRP on these cell-cell interactions offers new insights on therapeutic regimes, speaking of stabilization of pCRP in its inert state (16). For example, in vascularized composite allografts (VCA), consisting at least in part of skin, T cells constitute the leading cell type in inflammatory infiltrates during rejection (55). In acute rejection, predominantly TH1 cells and cytotoxic T cells are present (56), and the rejection process is orchestrated by CRP, whereas stabilization of pCRP via 1,6-bis PC reverses this effect (32). This understanding could furthermore be beneficial to autoimmune diseases like rheumatoid arthritis or solid organ transplantation.

Notably, isolated T cells seemed to have a higher activation in general when compared to PBMC or whole blood. This might be caused by the process of magnetic sorting itself, which is known to influence cells in their activation state and functionality (21, 57, 58).

The production of IL-17A was generally very low, which can be attributed to the fact that the cells were not further polarized by the addition of supplements. The primary amount of TH17 cells is, as shown in **Supplementary Figure 2**, relatively small.

In conclusion, this study contributes valuable insights into the complex and nuanced interactions between CRP and T cells, emphasizing the importance of various factors, including CRP confirmation and configuration, cell type and their activation status, as well as cells’ environment. We propose that mCRP-mediated T cell activation occurs via a monocyte-dependent cell-cell-contact involving the CD80/CD28 pathway. The findings presented here may pave the way for targeted therapeutic interventions in immune-related disorders.

## Data Availability

The original contributions presented in the study are included in the article/**Supplementary Material**. Further inquiries can be directed to the corresponding author.
