# Supplementary material for: C-reactive protein induced T cell activation is an indirect monocyte-dependent mechanism involving the CD80/CD28 pathway
Source: Front Immunol. 2025 Jul 18;16:1622865. doi: 10.3389/fimmu.2025.1622865 (PMC12313514; doi:10.3389/fimmu.2025.1622865)
Supplement: Supplementary file 1 [file DataSheet1.docx]

Supplementary Material


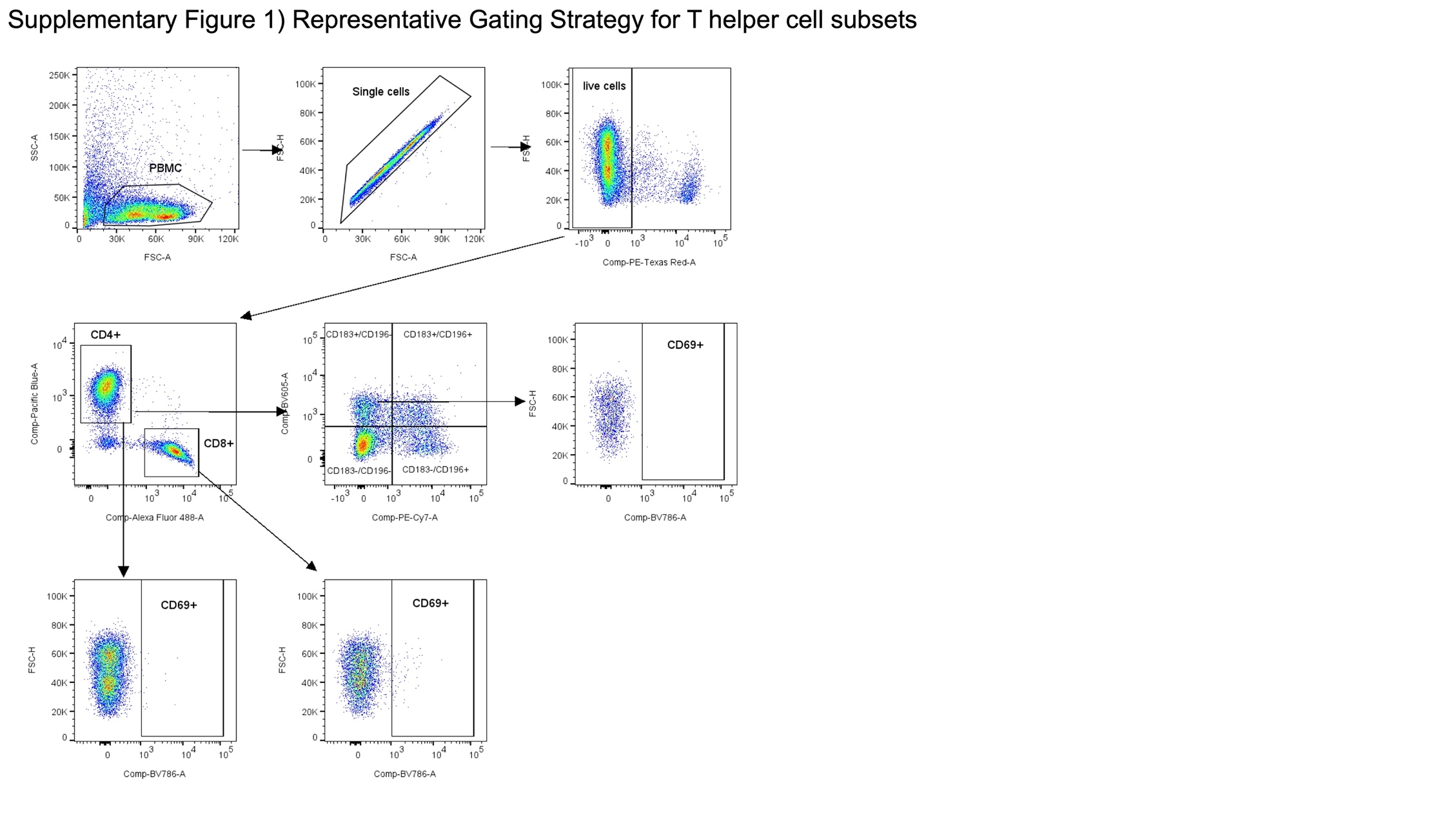


**Supplementary Figure 1. Representative Gating Strategy for T helper cell subsets.** T cells were identified based on their size and granularity (FSC/SSC), along with exclusion of dead cells. We further classified into CD4+ T helper cells and CD8+ cytotoxic T cells. CD4+ helper cells were subdivided into Th1 (CD183+ CD196-), Th2 (CD183- CD196-), Th17 (CD183- CD196+) and Th17-1 (CD183+ CD196+) based on the expression of CD183 and CD196. T cell activation was evaluated by measuring CD69 expression with FMO-controls used for appropriate gating and correction. Here, we show the control group without any supplemented CRP isoform.


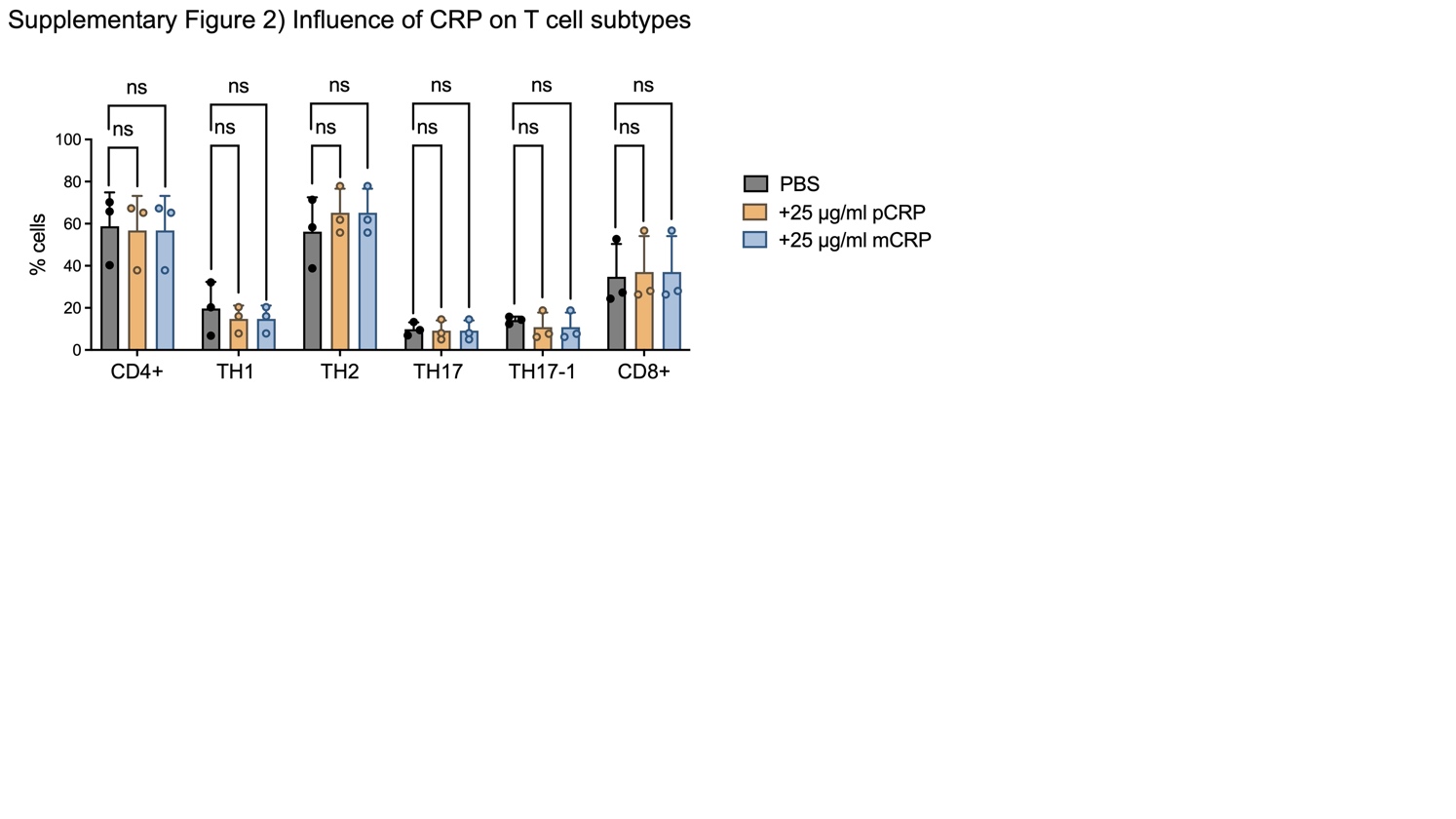


Supplementary Figure 2. Influence of CRP on T cell subtypes. PBMC were incubated for 7 days at 37°C and 5 % CO_2_. 25 µg/ml pCRP and mCRP were added respectively. T cell subtypes were differentiated by expression of surface markers CD4, CD183, CD196 und CD8 as followed: TH1 cells (CD183+ CD196-), TH17-1 cells (CD183+ CD196+), TH2 cells (CD183- CD196-), and TH17 cells (CD183- CD196+). Results are shown in mean and standard deviation. n=3. ns = not significant.


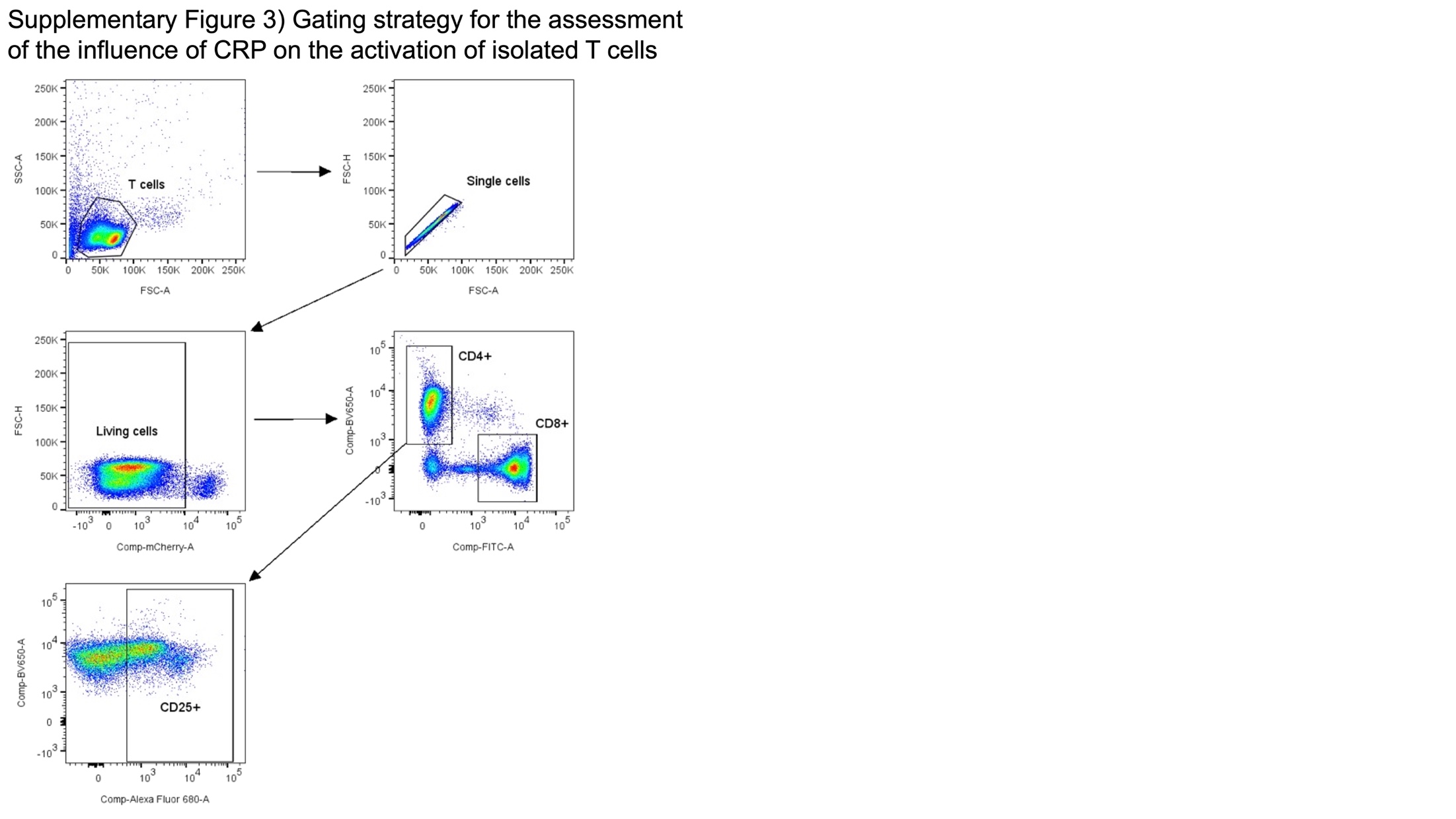


**Supplementary Figure 3.** Representative Gating Strategy for assessment of the influence of CRP on the activation of isolated T cells. T cells were sorted using magnetic beads and then incubated with PBS, 25 µg/ml pCRP, and 25 µg/ml mCRP for periods of 3 and 7 days. The T cells were identified based on the forward and sideward scatter, and duplicates were excluded from analysis. Activation was assessed for CD4+ and CD8+T cells by measuring the expression of CD69 and CD25. This figure panel illustrates the gating strategy without stimulation and CD25+ as an example.


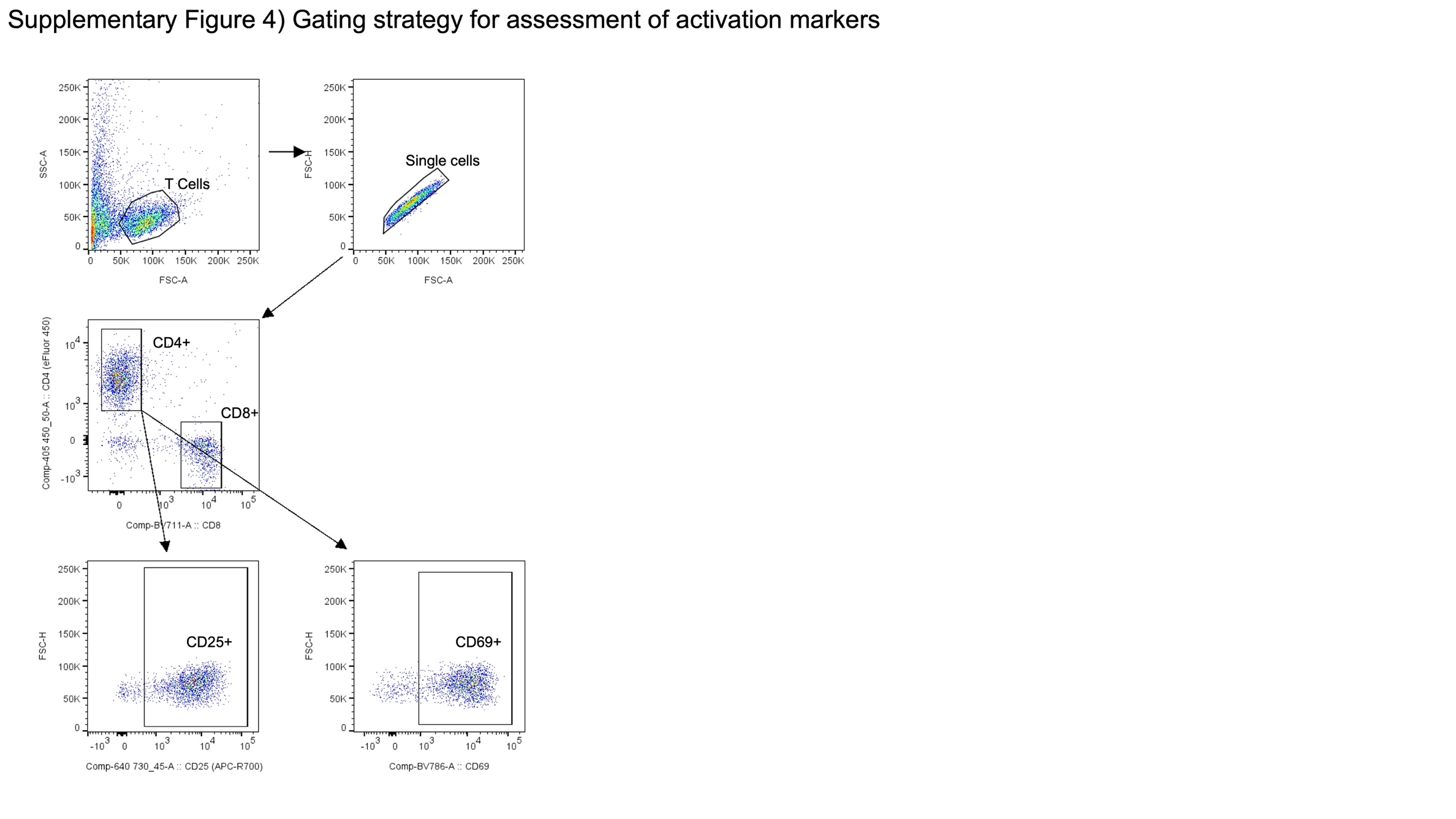


**Supplementary Figure 4.** Gating strategy for assessment of activation markers. T cells previously sorted by magnetic beads were incubated with 25 µ**l**/ml CD3/CD28 beads (1:1 bead number:cells) at 37°C and 5 % CO_2_ for 6 h, 24 h, 48 h and 5 d supplemented with 50 µg/ml pCRP or mCRP. T cells were located in forward/sideward scatter, duplicates were excluded. Subtypes were distinguished with CD4+ and CD8+. CD69+ and CD25+ activation was assessed for CD4+ and CD8+ T cells individually. This figure panel demonstrates the gating strategy utilized, using 24 h CD3/CD28 beads only control and CD4+ cells as an example.


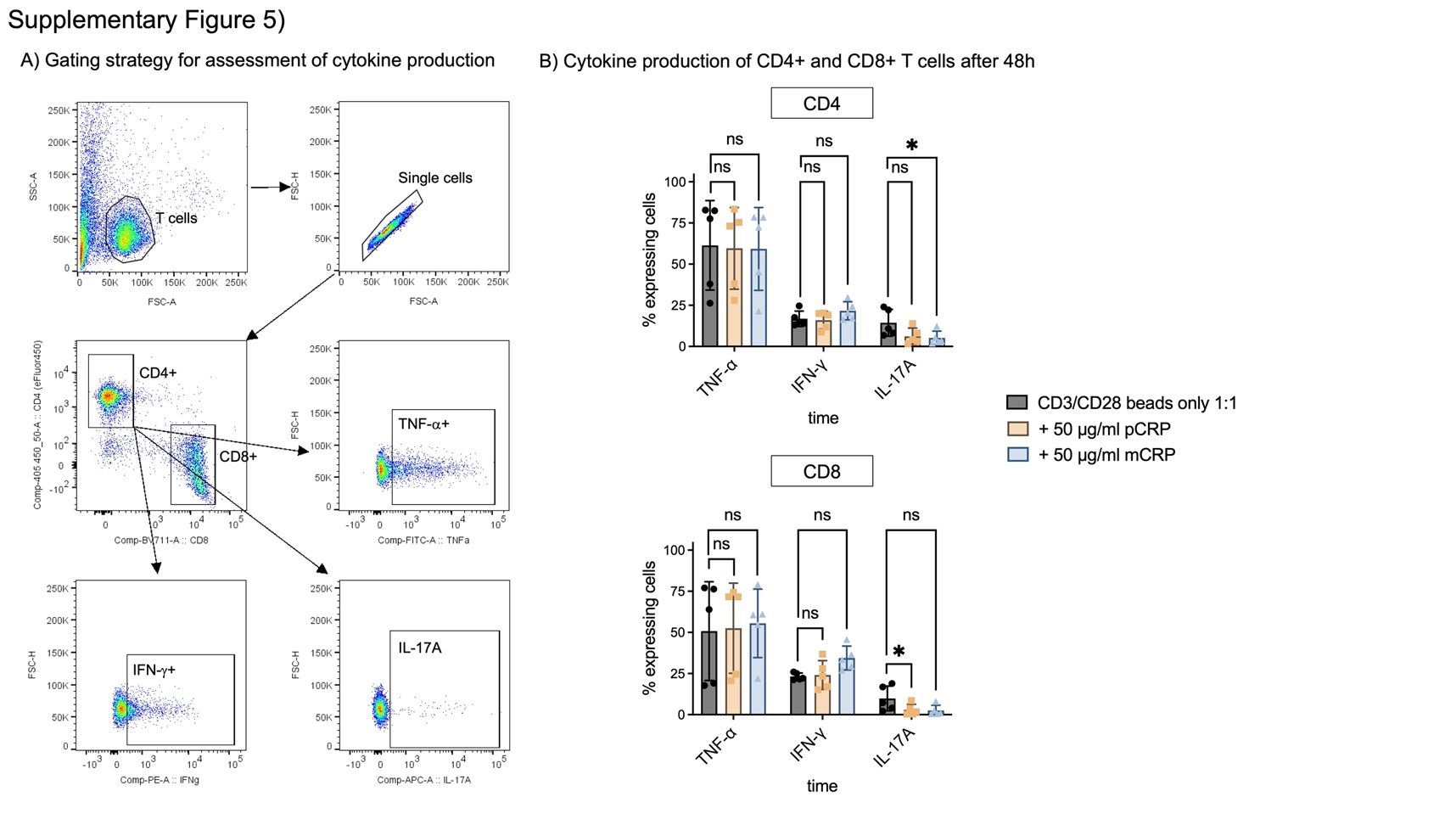


Supplementary Figure 5. (A) Gating strategy for assessment of cytokine production. Flow cytometric data was analyzed in FlowJo. Isolated T cells were located in forward/sideward scatter, duplicates were excluded. Subtypes were distinguished with CD4+ and CD8+. CD45RA served as naivety marker. Cytokine production in terms of TNF-α, IFN-γ, and IL-17A expressing cells was assessed after 48 h for CD4+ and CD8+ T cells individually. (B) Cytokine production of CD4+ and CD8+ T cells after 48 h. The curve displays the amount of TNF-α, IFN-γ and IL-17A expressing cells after 48h when incubated with CD3/CD28 beads only or additionally 50 µg/ml pCRP or mCRP. All results are displayed as means and standard deviation. n=5. * p<0.05, ns = not significant.


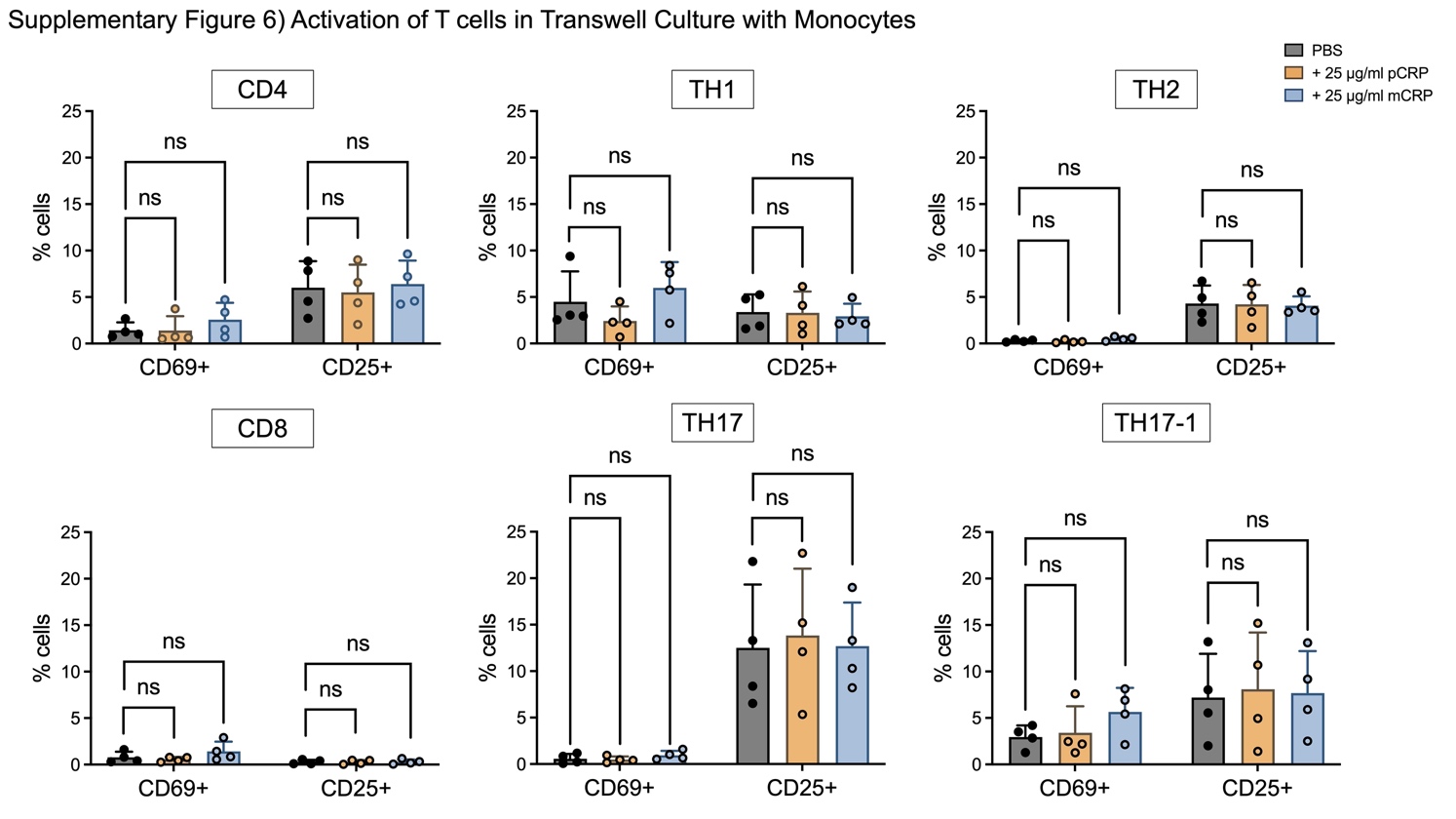


Supplementary Figure 6. Activation of T cells in Transwell Culture with Monocytes. T cells and monocytes were isolated from the same donor and separated by a 0.4 µm cell culture insert. Cell-cell contacts are not possible. However, cytokines and other molecules can circulate freely. Both cell populations were supplemented with 25 µg/ml pCRP or mCRP and incubated at 37°C and 5 % CO_2_ for 3 days. Results are shown in mean and standard deviation. n=4. ns = not significant.
